# Family-based tests applied to extended pedigrees identify rare variants related to hypertension

**DOI:** 10.1186/1753-6561-8-S1-S31

**Published:** 2014-06-17

**Authors:** Mengyuan Xu, Harold Z Wang, Wei Guo, Haide Qin, Yin Y Shugart

**Affiliations:** 1 Division of Intramural Research Program, National Institute of Mental Health, National Institutes of Health, Building 35, Room 3A 1000, 35 Convent Drive, Bethesda, MD 20892, USA

## Abstract

The application of family-based tests to whole-genome sequenced data provides a new window on the role of rare variant alleles in the etiology of disease. By applying family-based tests to these data, we can now identify rare variants associated with disease. Approaches for common variants, by contrast, require large sample sizes for power, and are powerless when faced with rare variants. When we tested Yip et al's 2011 family-based association tests for rare variants on pedigrees from the Genetic Analysis Workshop 18, we found that weighted collapsing methods generally have more power than unweighted methods, but are more prone to type I errors. We then evaluated a sliding window modification of the weighted family-based association tests for rare variants method. Although this modification inflates the rate of false positives, it significantly increases the power of family-based association tests for rare variants to identify causal rare variants.

## Background

Until next-generation sequencing (NGS) technology became widely available, genetic studies, including genome-wide association studies (GWAS), were generally based on the assumption that common diseases are driven by common variants (CD-CV). Although studies based on the CD-CV approach have had some success in identifying variants related to genetic disorders, their performance with regard to more complex diseases (such as hypertension or various psychiatric conditions) has been limited at best [1]. Researchers today recognize that most common disorders may result from the aggregate effects of multiple rare variants [2].

Because disease-related variants are likely to be both widespread in distribution and rare in frequency, GWAS data, which covers only a small portion of the genome, may not provide a representative sample in which to find them. By contrast, whole genome sequencing (WGS) technologies not only provide more complete coverage of the genome, but also of individual variants within genes. New ways to analyze and interpret WGS data are needed, however, because WGS is still costly, sample sizes are relatively small, and the disease-related variants are rare. Toward that end, family-based association test (FBAT) analyses, which enrich the occurrence of rare variants within pedigrees, provide a new approach to identify rare and possibly disease-related variants. This study shows how well the pedigree-based FBAT-rare method developed by Yip et al [3] performs when applied to WGS data from extended families for the identification of rare causal variants underlying complex traits such as blood pressure.

## Methods

### Data description

In this study, 2 FBAT-rare methods of statistical analysis were tested on the Genetic Analysis Workshop 18 (GAW18) data set based on the San Antonio Family Studies. GAW18 data includes 1389 individuals in 20 large pedigrees, with a maximum of 5 generations per pedigree. Subjects are randomly selected low-income Mexican Americans and their extended pedigree members. Study probands range in age, on average, between 40 and 60 years. The genotype data is cleared of mendelian errors on odd-numbered chromosomes, and comes from 959 individuals. Of these, 464 were sequenced at 60× coverage. Data from the rest were imputed. In total, the genotype data used in this study included 8,348,674 variants. For the purpose of assessing statistical methods, a set of genes were selected and their effect sizes calculated based on their correlation with hypertension, as well as their PolyPhen scores; 200 replicates of phenotypes were simulated. This set of genes is listed in the GAW18 answers. For convenience, we refer to the genes used to simulate phenotypes as true causal genes and the rest as noncausal. With this information, we assessed the power and false-positive rates of FBAT-rare, [3]. The method itself is not affected by our knowledge of the answer.

### Analytical Methods

#### Family-based association tests for rare variants (FBAT-rare)

In collapsing methods of statistical analysis, the effects of individual rare variants are combined linearly and the resulting sum is treated as a single variant. Many studies have been conducted to develop collapsing algorithms powerful enough to detect rare variants [4]. FBAT-rare tests, however, are among the first to apply collapsing methods to the analysis of pedigree-based data [3].

In this study, we investigated 2 different weighting schemes for test statistics implemented in the FBAT software (FBAT v2.0.4 beta 1). Under the FBAT-v0 test, the weights are defined as: 1, that is $w_{k}=1,k=1,...,M$, whereas under the FBAT-v1 test, the weights are defined as: $w_{k}=1/\sqrt{\zeta p_{k}\left(1-p_{k}\right)},k=1,...,M$, where *ζ *is the total number of nuclear families and $p_{k}$ is the allele frequency for the *k*th variant estimated from the sample. This weight scheme follows that used by Madsen and Browning [5] to estimate allele frequencies in controls sampled from the general population. (Note that both FBAT-v0 and FBAT-v1 are 2-sided tests that have now been implemented in FBAT v2.0.4 beta 1.)

Both methods were tested for each gene on chromosome 3 and repeated 200 times for each replicate, resulting in 200 *p *values for each gene. We observed that these *p *values often differ from each other by several orders of magnitude, preventing us from averaging all 200. Thus, we needed appropriate measures to compare different genes. To achieve this, we looked at a decreasing series of powers of 10. For an arbitrary gene, we calculated the fraction of *p *values that falls below 1*$10^{-i+1}$ (Table 1) and defined this as the power of finding the gene at the threshold of $10^{-i}$. Then, we find the smallest $10^{-x}$, such that this fraction is strictly above 0.5, and call it the power of both tests, to simplify comparisons.

**Table 1:** Power of FBAT-rare methods by *p *value of true-positive variants detected

Significance level	*p *Value threshold			
	
	$10^{-7}$	$10^{-6}$	$10^{-5}$	$10^{-4}$
**Genes analyzed**	Power			

***MAP4***				

**v0**	0	0	0.125	1

**v1**	0.005	0.955	1	1

**v0 corrected**	0	0	0.12	1

**v1 corrected**	0.03	0.94	1	1

***SCAP***				

**v0**	0	0	0	0

**v1**	0	0.005	0.975	1

**v0 corrected**	0	0	0	0.015

**v1 corrected**	0	0.085	1	1

***ARHGEF3***				

**v0**	0	0	0	0

**v0 corrected**	0	0	0	0

**v1 **	0	0	0	0

**v1 corrected**	0	0	0	0

## Results

### FBAT-rare methods

This study focused solely on chromosome 3. We defined genes according to the hg19 database (http://hgdownload.cse.ucsc.edu/goldenpath/hg19/database/). To highlight the statistical properties of rare variants, we selected variants with minor allele frequencies (MAFs) of <0.01, based on the founder population, for each gene. Using both v0 and v1 methods, we collapsed rare variants for each gene into a single variant. Both methods were then applied to all 200 replicates of simulated phenotype data, with the focus on the SBP_1 trait, which is the first systolic blood pressure measurement out of 4. Possible effects of sex, age, and medication on SBP_1 were taken into account to generate a corrected ASBP_1 phenotype, which was then analyzed in the same way as the uncorrected SBP_1 phenotype.

**Figure 1:**
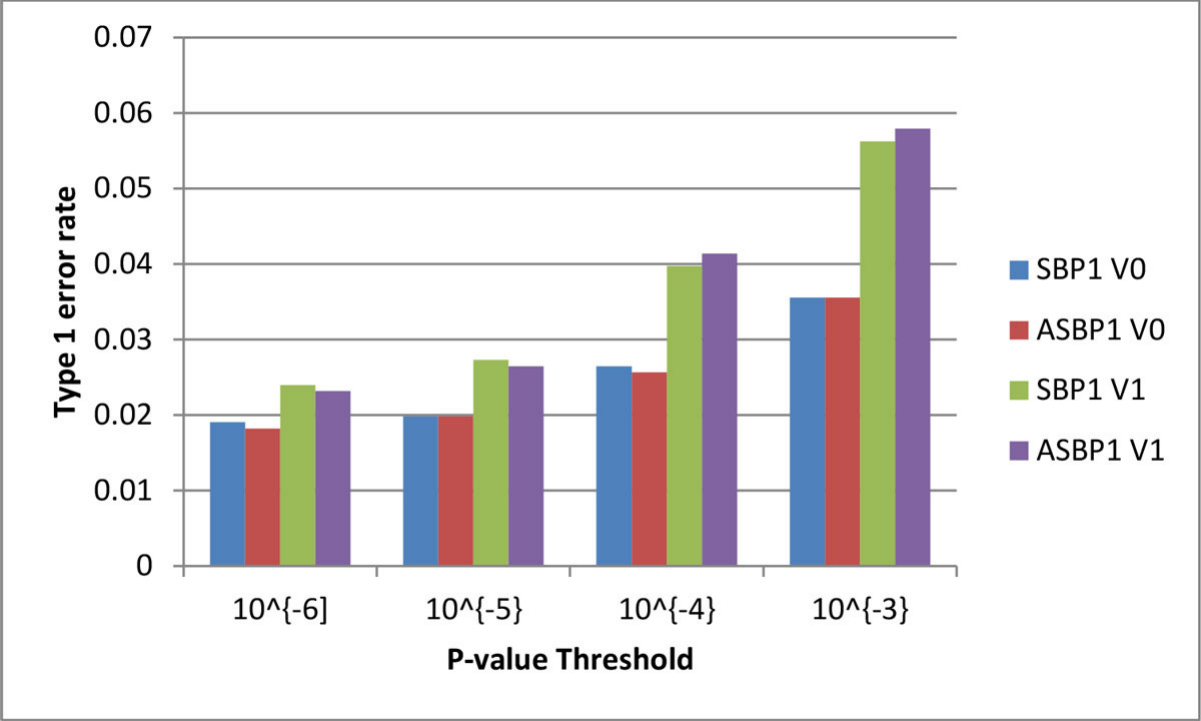
**Type I errors generated by FBAT-v0 and FBAT-v1 analysis of adjusted and unadjusted phenotypes at 4 different *p *value thresholds**.

Our assessment of the FBAT-rare methods' power to detect causal variants and their susceptibility to type I error is based on the *p *values derived, as described below, for all genes. In the first task, the power of FBAT-rare methods (calculated as the proportion of collapsed variants with *p *values within the range of significance) was tested using true causal genes from chromosome 3 (see Table 1). The rate of type I errors was calculated using noncausal genes (Figure 1).

Of the true causal genes, collapsed rare variants within *MAP4 *gave the strongest signal for significant association with the phenotype of interest. Both FBAT-v0 and FBAT-v1 methods detected these association signals, with *p *values of less than $10^{-4}$, in *MAP4*. In the *SCAP *gene, by contrast, only the v1 method detected associations at this level of significance, and neither method detected associations in other causal genes (data not shown, except for *ARHGEF3*). Based on our results for all causal genes, it is clear that the v1 method consistently outperforms the v0 method and is more sensitive than v0 for the detection of causal variants. In our data, if the *p *value for v1 falls below 0.1, then the v1 test consistently performs better than v0 for all cases tested.

To assess the validity of both methods, we selected all noncausal genes from chromosome 3 (using the same procedures that were used to calculate the power of causal genes) to calculate the probability of chance findings. See Figure 1 for the results, using v0 and v1 methods, with both the corrected and uncorrected phenotypes.

At the significance threshold *p *value of $10^{-3}$, both FBAT-rare methods identify fewer than 6% of significant associations in the 1209 noncausal genes. When the number of false positives decreases as the threshold becomes more stringent, even at the $10^{-6}$ threshold, the FBAT-rare methods identify significant associations in 2% of the noncausal genes. In addition, the v1 method, which is definitely more sensitive than the v0 method, is also unambiguously more prone to identifying false positives. Whether the phenotype is adjusted or unadjusted, though, does not appear to affect type I error rates.

### Sliding-window approach

That of 31 true causal genes simulated on chromosome 3, only *MAP4 *and *SCAP *were detected at the $10^{-4}$ significance level, indicates that neither the FBAT-v1 nor the FBAT-v0 method has ample power to detect rare variants. Because small genes are easier to detect than large genes, however, we decided to compensate for gene size using a sliding-window approach.

**Figure 2:**
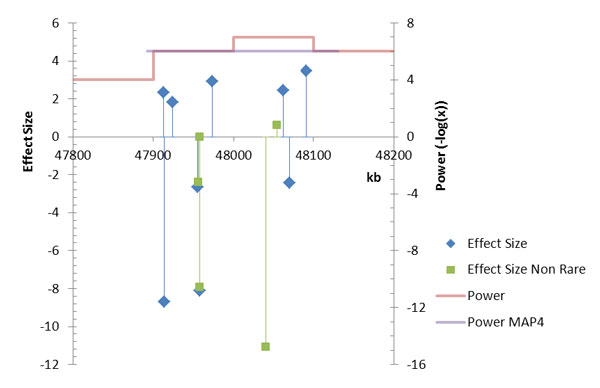
**Performance of sliding window analysis of *MAP4 *gene**. Blue and green drop lines show the positions of causal variants on *MAP4 *and the effect size for the adjusted systolic blood pressure trait. The green line represents the actual *MAP4 *region. The red line indicates the position of the 4 sequential windows, and its height shows the power of each window to detect rare variants. The purple line represents the power attained by analysis of *MAP4 *as a whole.

First, we divided the entire chromosome into a series of disjointed 100-kilobase (kb) windows with the whole 100kb points on the genetic positions (0-100 kb, 100-200 kb,...) as the starting and ending points. Using *MAP4 *as an example, the entire genetic region spans across 4 such windows. The reasoning is that any clustering of causal variants to fall within such a window will show up prominently. We then tested for rare variants using FBAT-v1 on data from each window, in the same manner as we tested the 2 FBAT methods on individual genes. *MAP4 *results (Figure 2) show that while the 47800 to 47900 kb and 48100 to 48200 kb windows contain data from a portion of the *MAP4 *region, they do not contain any of the causal variants. The 48100 to 48200 kb window still exhibits a significant signal in the p = $10^{-6}$ region, for a result that is as good as that achieved when FBAT-v1 is applied to *MAP4 *as a whole. Although the 47800 to 47900 kb window contains no signals of that strength, it reached a respectable power of $10^{-4}$, which can be partially explained by the high level of intermarker linkage disequilibrium between the 2 regions and *MAP4 *(data not shown).

For windows completely contained within *MAP4*, FBAT-v1 detected variants in the 47900 to 48000 kb and 48100 to 48200 kb windows with the same power as the gene-based method, and in the 48000 to 48100 kb window, detection even reached a power of $10^{-7}$, a whole magnitude better than a gene-based analysis of *MAP4 *can achieve. With most causal genes, results from FBAT-rare v1 analysis of the best window are an order of magnitude better than the results from gene-based analysis. The sliding-window approach, however, tends to result in more false positives (data not shown).

Because we used windows with a fixed length of 100 kb, the number of tests we did varies by the length of the gene. The *MAP4 *gene for example, spans 4 windows. Larger genes spanning more windows would be subjected to more tests, whereas smaller genes spanning only 1 window are tested only once. In genes that span more than 1 window, multiple testing becomes an issue. In the case of *MAP4*, because the gene's 9 rare causal variants all fall within the 2 windows (see Figure 2), using Bonferroni correction would effectively double its *p*-values. When we applied the Bonferroni correction, the power of the best performing window at $10^{-7}$ fell from 0.745 to 0.455. Both, however, far outclass the power of the *MAP4 *gene evaluated as a whole, which stands at 0.005 at the threshold of $10^{-7}$. Even if more stringent corrections were applied, the sliding-window approach would still have better power than the gene-based approach. Therefore, in the case of *MAP4*, correcting for multiple testing does not erase the power gain resulting from the sliding window approach.

## Discussion

In this study, we analyzed the GAW18 data set using 2 FBAT-rare methods (v0 and v1) to assess their power to detect rare causal variants associated with complex diseases. Results from these tests show that the weighed v1 method has better detecting power than the v0 method (see Table 1) but is more prone to type I errors (see Figure 1). With both methods, the only 2 positive hits for true causal genes to reach a level of significance were in the *MAP4 *(p = 10^−6^) and *SCAP *(p =10^−5^**) **genes. Both methods failed to detect the other simulated true causal genes at more than a 10^−4 ^level of significance. Both suffer from elevated type I error with roughly 6% of hits reaching levels of high significance (*p *value of 10^−3^).

We noticed that larger genes were more likely to be penalized when using the collapsing method. To see if we could improve these results, we reanalyzed the data with the FBAT-v1 method using a sliding-window approach. Our reasoning is that since the result for the collapsing method depends largely on the relative proportion of causal rare variants within a gene, breaking large genes into several windows would remove the diluting effect that regions with no causal variants have on the power of association tests.

Our results indicated that the sliding window approach did improve the v1 method's performance in detecting causal rare variants for large genes. However, when genes are broken down into multiple windows, the question arises whether the power gain is an illusion caused by multiple testing. To address this concern, we applied Bonferroni correction on the best-performing window of *MAP4*, and found that although the power decreased compared to the uncorrected results, it is still significantly higher than what *MAP4 *as a whole achieved. This confirmed that at least in the case of *MAP4*, the power improvement resulting from the sliding window approach was real. However, multiple testing corrections would be harsher for large genes that span many windows. Therefore, we do not dispute that in some cases the power gain when using the sliding window approach is a result of multiple testing alone.

We note that the relatively modest performance of the 2 FBAT-rare methods may reflect the limited size of our sample and the rarity of minor alleles. The fact that many of the simulated variants had only a modest effect does not help to distinguish them from background noise, and information is lost when the FBAT software breaks large pedigrees into nuclear families. Finally, linkage disequilibrium could have led to inflated numbers of false positives.

Our future studies will focus on comparing the FBAT-v0/v1 with alternative methods such as Betafam, proposed by Guo and Shugart [6], as well as developing novel methods that preserve the structure and thus the information contained in complex pedigrees.

## Conclusions

We tested 2 family-based association tests for rare variants (FBAT-rare) on 20 pedigrees provided by the GAW18, and our results indicated that weighted collapsing methods have more power than unweighted methods and inflated type I errors. We also applied a sliding-window approach to the same data sets. Although that approach tends to inflate the false-positive rates, it does significantly increase the power to identify some of the causal rare variants in GAW18 data.

## Competing interests

The authors declare that they have no competing interests.

## Authors' contributions

YYS, MX, and WG conceived of and designed the study. HZW, MX, and HQ performed data annotation and analysis. The manuscript was analysed by MX and written by MX, HZW, YYS, WG and HQ.

All authors read and approved the manuscript.
